# Improving identification of differentially expressed genes in microarray studies using information from public databases

**DOI:** 10.1186/gb-2004-5-9-r70

**Published:** 2004-08-26

**Authors:** Richard D Kim, Peter J Park

**Affiliations:** 1 Harvard-Partners Center for Genetics and Genomics, 77 Avenue Louis Pasteur, Boston, MA 02115, USA; 2 Children's Hospital Informatics Program, 300 Longwood Ave, Boston, MA 02115, USA

## Abstract

We demonstrate that the process of identifying differentially expressed genes in microarray studies with small sample sizes can be substantially improved by extracting information from a large number of datasets accumulated in public databases. The improvement comes from more reliable estimates of gene-specific variances based on other datasets. For a two-group comparison with two arrays in each group, for example, the result of our method was comparable to that of a *t*-test analysis with five samples in each group or to that of a regularized *t*-test analysis with three samples in each group. Our results are further improved by weighting the results of our approach with the regularized *t*-test results in a hybrid method.

## Abstract 2

The process of identifying differentially expressed genes in miroarray studies with small sample sizes can be improved substantially by extracting information from a large number of datasets accumulated in public databases.

## Background

Microarray experiments are often used to identify potentially relevant genes in biological processes. By determining which genes are differentially expressed between different states, for example, hypotheses can be developed as to the role of those genes in the underlying biological mechanism [1-4]. However, the fact that microarrays simultaneously assess the expression of tens of thousands of genes makes it difficult to extract pertinent information from background noise. With a multitude of variables, it is easy to generate a high percentage of false positives, and validation is expensive and time-consuming. This issue is aggravated by the high cost of microarrays and often by the difficulty of obtaining enough biological or clinical samples, causing microarray experiments to be performed on a smaller scale than desirable in almost all cases. For exploratory analysis in particular, very few biological or technical replicates are run at present. For a two-class comparison, three-by-three or smaller experiments are not uncommon. For brevity, we will use the notation 'NvN' or 'N by N' to denote a two-group comparison with N arrays vs N arrays.

Overall, the need for a large sample size is acute for expression profiling studies. The number of arrays needed in a study depends on many factors, including the study design, the magnitude of biological variation in the samples, technical variability introduced in the experiment, and the desired level of sensitivity and specificity for differential expression. Several studies have examined this issue. A model with additive and multiplicative noise was used to derive the number of samples necessary for detecting fold changes of given magnitude when false-positive and false-negative rates are specified [5]. The difficulty, however, is that parameters describing technical and biological variations must be estimated for the model, which is not an easy task. When 16 public datasets, mostly from cancer studies, were examined using a repeated sampling approach [6], it was observed that stable results for differentially expressed genes are not obtained until at least five biological replicates are used and that 10-15 replicates are needed for sufficient stability. This is consistent with the results obtained in [7]. According to these criteria at least, many microarray studies are vastly underpowered. From the perspective of analysis, it is always desirable to have sufficient data. Some data analysts may even insist on a minimum number of samples before starting statistical analysis. However, when practical considerations limit the sample size, it is important to work with the given data in an optimal manner to extract as much information as possible.

In the context of finding differentially expressed genes, the null hypothesis for each gene is that it is not differentially expressed between two groups, usually against the two-sided alternative hypothesis that the gene is up- or downregulated. The most commonly used statistical test in this setting has been the two-sample *t*-test, although other similar statistics such as the signal-to-noise ratios [1] have often been used as described below. There are a variety of statistical issues involved with identifying differential expressed genes, such as the adjustment of *p*-values for multiple testing [8] and the use of the false-discovery rate [9]. Ideally, the joint distribution of the test statistics should be considered, in order to account for correlation among the genes [10], but in practice, because of the difficulties associated with the number of genes being many times that of the samples, most testing procedures are carried out in a univariate manner for each gene [11]. The method we introduce here also performs a test independently for each gene and ignores correlation among genes.

A fundamental difficulty in drawing reliable conclusions from a small number of samples lies in accurate estimation of the gene-specific variances, or the variance of a difference in mean expression levels per gene, with which to determine the statistical significance of observed changes in expression. Because variances based on a very small number of samples tend to fluctuate wildly as a result of randomness in sampling from a population, our ability to assess differential expression is drastically impacted. A naive application of standard methods used for larger sample sizes can result in a large number of false positives for differential expression. For example, with a small sample size, the list of significant genes identified by the *t*-test or variations thereof is crowded by a large fraction of genes for which large *t*-statistics are due to underestimation of variance by chance.

Many methods have been devised to address this problem. A popular approach has been some type of regularization of the *t*-test. A Bayesian framework for combining the variance estimate with a background variance associated with neighboring genes was developed in [12]; a method of pooling errors among genes in which expression values are similar is presented in [13]. In the popular significance analysis of microarrays (SAM) method, a small constant is added to the variance estimate to prevent it from getting too small [14]; empirical Bayes methods compensate for the lack of enough replicates by combining information across the arrays [15-17]. Nonparametric methods [18], analysis of variance approach [19], and Bayesian hierarchical models [20,21] are also available. Some of these methods are compared in [22].

Whereas all the available methods attempt to improve the identification of differentially expressed genes essentially by gathering information across similar genes, we suggest another solution. We propose estimating the natural variance of individual genes using a large number of experiments performed previously. This provides a different and potentially more stable and accurate estimate of the variance for each gene than by simply looking at the variance of a small number of expression levels, especially in studies with very small sample sizes. Using these variances as the basis for determining differential expression offers an alternative method that can reduce the false-positive rate significantly. As the most effective method, we propose a hybrid method in which we combine the variance estimate from the current dataset with the estimate from previous experiments. This approach can also be incorporated in other settings, especially in a Bayesian framework with prior distribution for variance derived from the database. It can be applied more generally to other testing procedures such as ANOVA that benefit from more accurate estimation of gene-specific variances, and can be easily extended to the estimation of the covariance matrix in multivariate analysis.

More reliable calculations of such variances based on many chips is becoming increasingly possible through large public databases of previous experiments. Public databases such as the Gene Expression Omnibus (GEO) [23] contain data from many chips, with the goal of gaining information from pooling data. GEO currently has thousands of chips, with a heavy skewing towards Affymetrix MG-U74Av2 and HG-U95 chips. Specifically, there are about a thousand HG-U95A chips and another thousand MG-U74Av2 chips, and these numbers are growing steadily (our gene-specific variances were calculated when the database held only 865 chips). Other large public databases include ArrayExpress [24], Yale Microarray Database [25], and Stanford Microarray Database [26]. GEO was selected as our primary source of reference because it had the largest compilation of single-channel microarray chips. We chose to analyze Affymetrix chips because the standardization of single-channel chips allows for easier cross-experiment comparison than dual-channel chips. The dual-channel chips are often custom-made and lack consistency in the genes represented; more important, different experiments use different reference channels, which makes it difficult to compare across experiments.

## Results

### Comparing various methods

We compared the performance of four methods in accurately assessing differential expression of genes: the standard *t*-test, the new GEO-adjusted method, the regularized *t*-test, and a hybrid method combining the GEO method and the regularized *t*-test. The primary difference between these methods lies in the denominator of each method's *t*-statistic. The GEO-adjusted method replaces the sample variance estimate in the denominator with the gene-specific variance calculated from the GEO database (details for the calculation of the variance, which can be either global or pooled, are described in Materials and methods). Hence, the genes are sorted using the modified *t*-statistic:



where *μ*_1*i*_, *μ*_2*i *_are the means for the groups 1 and 2, respectively, for the *i*th gene, *n*_1 _and *n*_2 _are the sample sizes in the groups 1 and 2, and *σ*^2 ^_*GEO*,1 _is the gene-specific variance from the GEO database.

The regularized method we used added a small constant to the denominator of the *t*-test, ranking genes based on the modified *t*-statistic:



where *σ*_0 _is the fifth percentile of all variances (*σ*_0 _can also be calculated to minimize the coefficient of variation of the statistic [14]). The regularized *t*-test smoothes out the effects of underestimated variances and therefore returns a more reliable assessment of differentially expressed genes in small samples than the standard *t*-test.

Finally, we introduce a hybrid algorithm that combines the GEO method with others through a voting mechanism. This provides a portable solution that can be combined with a variety of other testing procedures and could potentially improve the performance of any other algorithms designed to determine differential expression in experiments with small sample sizes.

### Testing procedure and dataset

To compare the effectiveness of the methods, we determined lists of differentially expressed genes in order of significance by applying each procedure to a large number of subsets of arrays of a given size. These genes were then compared with the 'master' list of differentially expressed genes to assess the accuracy of the method. Because we generally do not know the correct ordering of genes with differential expression, we substituted the list obtained by the *t*-test analysis for the full dataset as the master list; with a sufficiently large dataset, this master list is a close approximation to the true list. Thus, we used the large dataset to compute a true *t*-statistic for each gene and then treated random small subsamples of the arrays from the dataset as simulated observed datasets from which we could compute estimated ranks for small subsamples. Because exploring all realizations of possible subsets for a large full dataset would be prohibitively time-consuming (for example, more than 10^8 ^combinations of 3v3 subsets for our first dataset), we sampled repeatedly for subsets until we obtained convergent results. We then compared these lists of differentially expressed genes with the master list for overlaps or correlations in the orderings. Once the size of the subsets approaches the size of the full dataset, there can be a substantial overestimation in the overlap of genes, owing to the fact that the master list is generated using the dataset from which the subsamples are derived. However, this effect appears to be negligible in our simulations because of the large size of our full dataset and the small subsample sizes that are of our interest.

The dataset primarily used for testing was a prostate cancer dataset [27] that had 50 normal samples and 52 tumor samples, with follow-up tests performed on a smaller Duchenne muscular dystrophy dataset [28] to confirm results. In our subsampling process, a small number of patients are randomly selected from each group and a variety of methods were used to determine a list of differentially expressed genes. We are mainly interested in very small sizes of one to three samples per group. For concreteness, we focus on the results for 2v2 comparisons first, but we also describe 1v1 and 3v3 comparisons. Note that large datasets are utilized here solely for the purpose of evaluating the method and that the method is designed to be used for studies with small samples.

### Numerical results with a GEO-adjusted *t*-test

**Figure 1:**
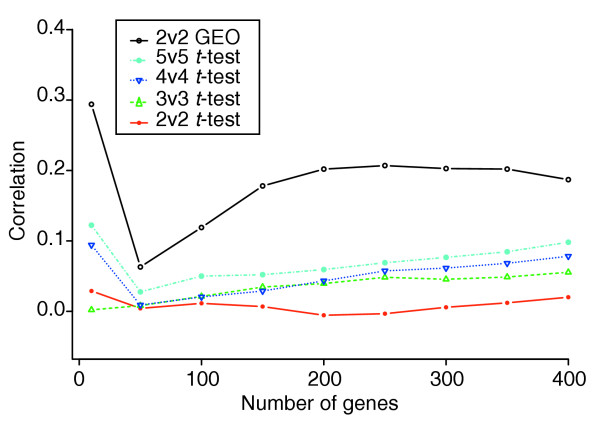
The correlation between the rank of the top genes with their 'true' rank, based on the 'master' list from the full data. The *x*-axis is the length of the gene list being compared. The correlation of the GEO method is clearly superior to the correlation of the simple *t*-test.

The first measure that was used to assess the effectiveness of the GEO-adjusted method was the correlation between the rank of the top genes returned by various methods and the true rank of those genes. This method was also used in [6]. In this measure, the standard *t*-test was compared to the GEO-adjusted method. The behavior of the correlation coefficient was tracked as the number of genes being analyzed was increased and the averaged values over many simulations are shown in Figure 1. If the method were perfectly effective and ranked genes in the same order as their true ranking according to the master list, the correlation should be 1. However, the correlation coefficients were surprisingly low. This reflects the great difficulty of obtaining accurate or stable lists of differentially expressed genes from small sample sizes. Nonetheless, Figure 1 reveals that the correlation improves for the *t*-test as the sample size increases, and that the results of GEO tend to correlate better with the master list than the results of 2v2, 3v3, 4v4, or 5v5 *t*-test.

**Figure 2:**
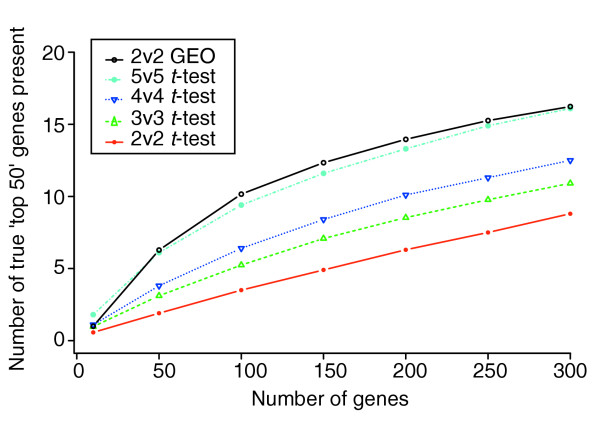
A comparison of the reliability of differential expression results returned by simple *t*-test and the GEO method. The number of the top 50 differentially expressed genes from the master list that are found in the gene list of length 10, 50, 100, 150, 200, 250 and 300 is indicated on the *y*-axis. The GEO results based on a 2v2 sample are comparable to the results returned by a 5v5 sample *t*-test.

To further assess the reliability of the results, tests were conducted to determine the number of top 50 genes from the master list that were accurately returned using various methods. In Figure 2, this is plotted as a function of the list length generated by these methods, at 10, 50, 100, 150, 200, 250 and 300. For example, a list of 100 genes from the 2v2 GEO method contains just over 10 genes from the top 50 genes from the master list. Again, the low overlap clearly illustrates the difficulty of obtaining an accurate list of significant genes. We believe the low numbers to be partly due to the nature of heterogeneous samples in our test datasets (see Discussion); therefore, we focus more on the trend among the various methods here. This metric indicated that the GEO method is considerably more reliable than the *t*-test at determining differentially expressed genes in small sample sizes. Compared to a simple *t*-test, the GEO method performs substantially better, returning results from a 2v2 test that are comparable to the results returned by a 5v5 experiment using *t*-test. Using GEO variances on a 2v2 test returns 231% more of the top 50 genes than the unadjusted *t*-test. While we are not suggesting that a simple *t*-test is a recommended method of assessing differential expression in such small sample sizes, it illustrates the potential of this method. Using gene-specific variances developed from GEO databases is clearly more accurate than the variances that an uninformed *t*-test derives from small samples. We do not plot the error bars for each measurement in the figures owing to space constraints, but we have verified in the important cases that the separation between the curves is significant.

**Figure 3:**
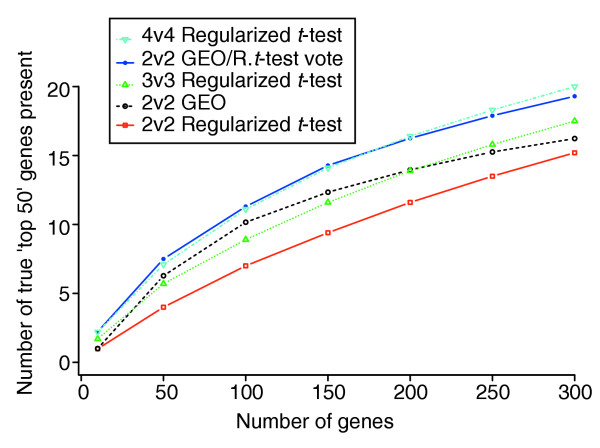
A comparison of the reliability of differential expression results returned by regularized *t*-test, the GEO method and the voting method in a 2v2 sample comparison. The number of the top 50 differentially expressed genes from the master list that are found in the gene list of length 10, 50, 100, 150, 200, 250 and 300 is indicated on the *y*-axis. The GEO results based on a 2v2 sample are clearly superior to the 2v2 regularized *t*-test results, and roughly comparable to the results of the 3v3. The voting method combining the results improves the results to a level almost comparable to a 4v4 regularized *t*-test.

The GEO-adjusted method also compared favorably to a regularized *t*-test. By smoothing out the variance estimates, the regularized *t*-test returns a more accurate assessment of differentially expressed genes than the standard *t*-test. Thus the gains from the GEO method over the regularized *t*-test were less substantial than over the standard *t*-test but still significant, especially for shorter gene lists. Improving our ability to reliably detect the differentially expressed genes with a short list is generally more valuable than doing so with a longer list simply because these genes at the top are the ones that an investigator examines most closely. As shown in Figure 3, the average gain of the 2v2 GEO sample versus the 2v2 regularized *t*-test in those three areas (50, 100 and 150 genes) was more than 30%. The performance of GEO on a 2v2 analysis seems roughly comparable to the performance of the regularized *t*-test on a 3v3 sample analysis.

### Superior performance of a hybrid method

One of the greatest advantages of the GEO method is that it can be combined with other methods. Because the regularized *t*-test and the GEO method both use different, yet effective, techniques to smooth out variance, they can both contribute to the differential expression analysis. By using a voting method that weights and averages the results returned from the regularized *t*-test and the GEO method, the performance improves further (see Materials and methods for details). The results of a 2v2 chip analysis using our voting method nearly match the performance of a 4v4 regularized *t*-test analysis, which is quite promising (Figure 3). As before, our incidence of the top 50 genes in the top 10 listed, top 50 listed and top 100 listed are improved. The voting method returns 88% more of the top 50 genes than the regularized *t*-test alone. We also see the greatest improvement in the larger sets of genes, thus negating one of the weaknesses of the GEO-adjusted method. By combining the advantages of the regularized *t*-test and the additional information from the gene-specific variances, we are able effectively to pare the required number of chips in this case and to elicit better results from the chips we do have. Further details are provided in the Materials and methods section.

**Figure 4:**
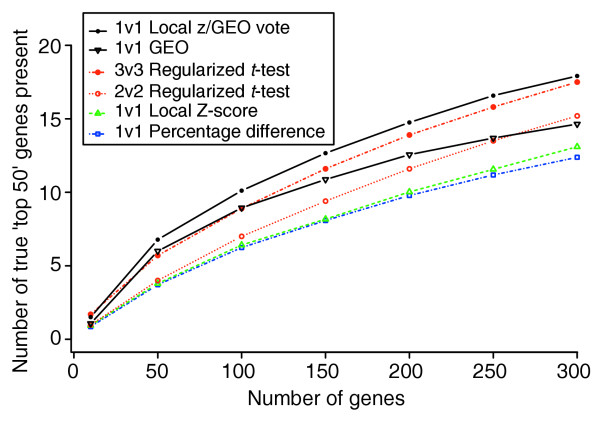
A comparison of the reliability of differential expression results returned by the GEO method, a few standard methods, and the voting method in a 1v1 sample comparison. The number of the top 50 differentially expressed genes from the master list that are found in a gene list of length 10, 50, 100, 150, 200, 250 and 300 is indicated on the *y*-axis. The GEO results based on a 1v1 sample are clearly superior to the 1v1 results from the local z-score method (as implemented in SNOMAD), or from the percentage difference (using percent changes relative to the mean expression), and almost comparable to the results of the 3v3 regularized *t*-test. The voting method combining the results improves the results to a level superior to a 3v3 regularized *t*-test.

Tests were also performed on other sample sizes, namely 1v1 and 3v3. Although we view the first case especially as an inadequate design and do not recommend it, we have found that investigators are sometimes forced to perform analysis on such a small number of samples. We are therefore interested in improving the effectiveness of such exploratory analysis, the results of which must be verified using other techniques such as quantitative reverse transcription PCR (QRT-PCR). In our 1v1 analysis, we compared the GEO method to three methods of ordering genes on the basis of differential expression: fold ratio, *y*/*x*; percent changes relative to the mean expression levels, (*x - y*)/((*x *+ *y*)/2); and z-score based on local variance correction (using locally weighted polynomial regression) across signal intensity, as implemented in SNOMAD [29]. Basic filtering of low expression was performed at the beginning. In the example dataset, the z-scores give slightly better results than the percent changes, which in turn were better than simple fold ratios. However, as shown in Figure 4, the GEO method returns 60% more of the top 50 genes than the best of the first two standard methods and generally returns superior results, almost on the same scale as a 3v3 regularized *t*-test. The method based on the z-scores performs slightly better than either of the standard methods, but GEO still returns 57% more of the top 50 genes. In the 1v1 case, the voting method proves useful, improving the results of both methods. By combining the z-score method and GEO's rankings, the results are superior to a 3v3 regularized *t*-test analysis. The voting method captures 83% more of the top 50 genes than the best of the standard methods. These results reflect the success of the voting method in combining GEO's rankings with a variety of other methods to significantly improve the overall performance.

**Figure 5:**
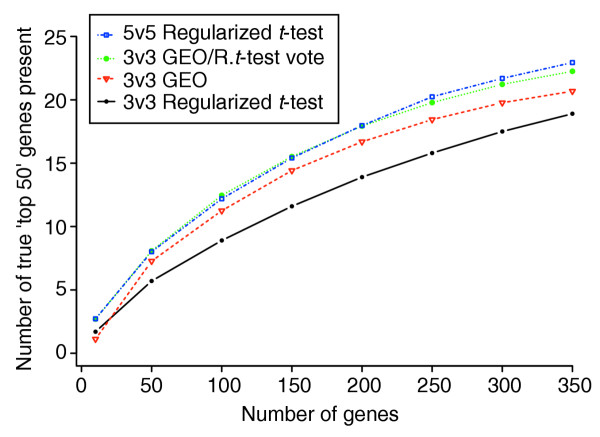
A comparison of the reliability of differential expression results returned by regularized *t*-test, the GEO method, and the voting method in a three sample by three sample comparison. The number of the top 50 differentially expressed genes from the master list that are found in the gene list of length 10, 50, 100, 150, 200, 250 and 300 is indicated on the *y*-axis. The GEO results based on a 3v3 sample are clearly superior to the 3v3 regularized *t*-test results, and roughly comparable to the results of the 4v4 regularized *t*-test (not shown). The voting method combining the results improves the results to a level almost comparable to a 5v5 regularized *t*-test.

The results from the regularized *t*-test and GEO method were also compared on 3v3 comparisons. Whereas GEO still returns more reliable estimates than the regularized *t*-test, the improvement is smaller than in the case of the smaller sample size comparisons. In the 3v3 case, the GEO-method results are comparable to those of a 4v4 regularized *t*-test, returning 17% more of the top 50 genes than the 3v3 regularized *t*-test. However, we do find that the voting method again improves the results, returning very similar numbers of correct genes as a 5v5 regularized *t*-test (Figure 5). The voting method returns 41% more of the top 50 genes than the 3v3 regularized *t*-test.

**Figure 6:**
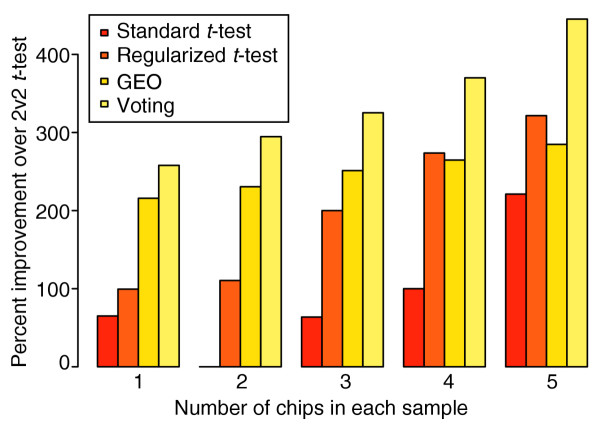
Summary of the performance of the four methods (standard *t*-test, regularized *t*-test, GEO method, and voting method). The bars indicate the percent improvement over the 2v2 standard *t*-test in identifying the top 50 differentially expressed genes. GEO performs better than the regularized *t*-test in smaller sample sizes, while the regularized *t*-test outperforms GEO in larger sample sizes. The voting method is substantially better in all cases.

The performance of the GEO method does not seem to be influenced greatly by the number of samples in each group. This is because the gene-specific variance estimates are fixed and adding additional samples only impacts the mean estimates for each group. In contrast, in the regularized *t*-test method, adding additional samples to each group refines the estimates of both the means and the variances of each group. This factor is the fundamental reason that the regularized *t*-test improves quickly as the number of samples is increased whereas the GEO method does not. However, GEO performs strongly even with only one sample in each population and generates results that are comparable to a 3v3 regularized *t*-test analysis. This indicates that the greater weakness in the small-sample *t*-test lies in inaccurate variance estimates, and that stable, accurate estimates of gene-specific variance can greatly improve analysis. These results are summarized in Figure 6, which compares the performance of the standard *t*-test, the regularized *t*-test, the GEO method, and the voting method across sample sizes. The voting method is substantially better in all cases.

**Figure 7:**
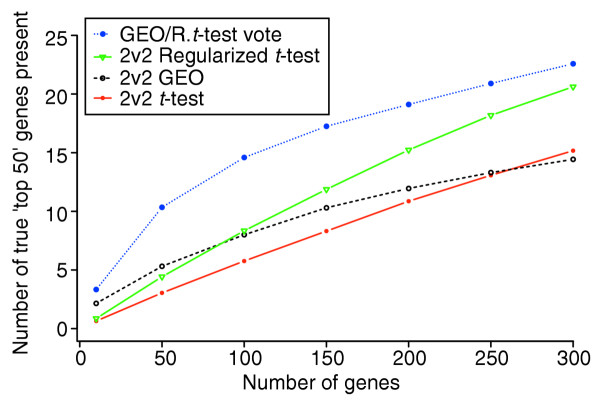
A comparison of each method in 2v2 subsampling of a Duchenne muscular dystrophy dataset. The most positive results are clearly seen in the voting method combining the regularized *t*-test and GEO results. This method returns 134% more of the top 50 genes than the regularized *t*-test alone and 240% more than the standard *t*-test.

**Figure 8:**
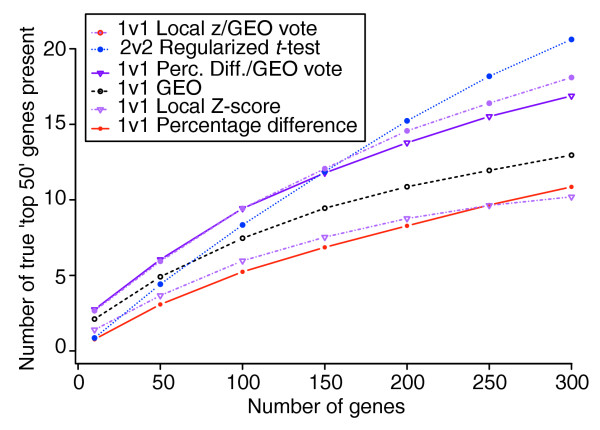
A comparison of each method in 1v1 subsampling of a Duchenne muscular dystrophy dataset. The most positive results are clearly seen in the two voting methods combining the GEO results with either the local z-score method (as implemented in SNOMAD) or the percentage difference method (using percent changes relative to the mean expression levels). These methods return 96% more of the top 50 genes than the standard method alone and 80% more of the top 50 genes within 100 genes.

Looking at the Duchenne muscular dystrophy dataset also provides us with corroboration of the usefulness of this method. In this situation, the dataset is much smaller (11 normals vs 12 DMD). Because two samples capture a much higher percentage of the data in 11 chips than in 50 chips, we expect the usual tests on subsamples to naturally provide results more similar to the master list. Therefore, we expect to see less of an improvement from GEO than in our cancer dataset. As before, we see the GEO results consistently providing better results in the smaller sets of genes than the standard *t*-test, returning 33% more of the top 50 genes in the 2v2 case (Figure 7) and 40-170% more of the top 10, 50, 100 and 150 genes in the 1v1 case (Figure 8). While the regularized *t*-test seems to outperform the GEO method, combining the results of both using our voting method again returns superior results. For example, averaging the ranks in the 2v2 case returns us 134% more of the top 50 genes than the regularized *t*-test alone and 240% more than the standard *t*-test (Figure 7). In the 1v1 case, the voting method clearly outperforms either the GEO method or the local z-score method (as implemented in SNOMAD) alone, providing results that seem roughly similar to those returned by a 2v2 regularized *t*-test analysis. These results indicate that, as shown in the cancer dataset, improved results can definitely be attained through incorporating gene-specific variance in differential expression analysis. Most important, because the GEO method can be combined with regularization methods through a voting procedure, it can be used to improve results regardless of how it individually performs on a dataset.

## Discussion

### Number of chips

**Figure 9:**
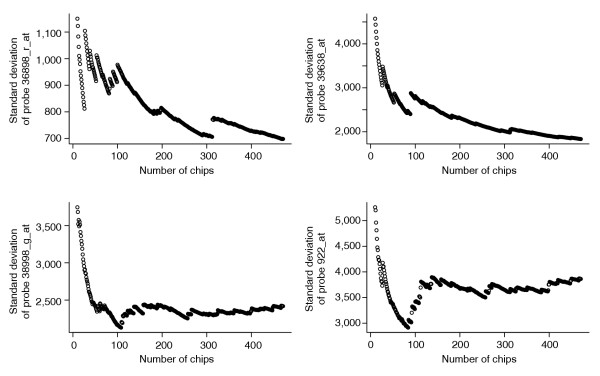
The progression of the variance estimate as the number of chips used in the estimation increases, for one realization of the chip ordering. After all the genes were sorted by their intensity level, one gene was selected from the middle of each quartile. As seen in each case, the variance calculated from many chips tends to converge as the number of chips grows. Generally, the variances seem to settle near their final values once 250-300 chips are gathered.

For this method to be successful, a significant number of previously run chips must be available. As public databases grow in size and number, this limitation will gradually diminish, but not all chip types currently have enough available chips to use this method. Whereas the most popular chip types (such as Affymetrix HG-U95A) have hundreds of previously run chips available, it is more difficult to find databases of the less popular ones. In an attempt to test for the number of chips sufficient to utilize this method, variance analysis was performed. In Figure 9, we plot the variance estimate as the number of chips used in the estimation increases, for one realization of the chip ordering. Because genes at different intensity levels may behave differently, we sorted the genes by their expression levels and selected four genes, one from the middle of each quartile. As seen in each case, the variance calculated from many chips tends to converge as the number of chips grows. Generally, the variances seemed to settle near their final values once 250-300 chips are gathered. After averaging over a large number of realizations in the chip order, we find that the variance settles near its final value at 250 chips, deviating less than 5% in either direction as more chips are gathered. While it is difficult at this time to find 300 chips of similar type and tissue, it should become easier to find datasets that are more specifically correlated with the experimental set as more data are accumulated in public databases. This would allow for more useful baselines to be established in calculating gene-specific variance, and would probably substantially improve the results.

### Comparing across multiple tissue types

When trying to estimate the gene-specific variances for a particular experiment, the best approximation would come from a database of similar experiments. Because gene expression profiles have the potential to vary widely in cell and tissue type, examining many other chips of the same tissue type should provide the best indication of the baseline variance. For example, it would make most sense to draw on a large database of cancer chips to derive relevant information for a cancer dataset. Unfortunately, because of the dearth of large datasets that match each other in tissue type, chip type, and post-processing, it is difficult at this time to test this theory. Because our public databases do not yet contain enough chips sorted by tissue type to perform this procedure, we are forced to mix all the chips of any given type together. Yet, even with only a database of totally unrelated chips, we still saw a significant increase in performance, even over already improved methods such as the regularized *t*-test. If our gene-specific variances were based on even more reliable estimates (such as samples from the same tissue or same disease), the performance of the GEO method would probably be increased. As public databases grow in size and organization, this should become increasingly possible.

### Comparing across multiple chip types

We have shown here how to derive more stable variances based on chips of the same type. The problem is much more complicated when multiple chip types are involved. In fact, we have observed that even different generations within the same platform do not give concordant results. When the same tissue samples were hybridized on both HG-U95A and HG-U133A chips, the dominant feature in the data was the chip type rather than the sample characteristic, and the lists of differentially expressed genes differed substantially between the two cases. Standardizing across these two types can be done for a portion of the genes but it is an involved process (Hwang KB, Kong SW, Greenberg SA, P.J.P., unpublished work). Efficiently combining data from single-channel and double-channel arrays is even more difficult. A more comprehensive database with a larger number of arrays spanning a greater variety of experiments would alleviate the problem to some extent, but methodologies for integrating data from multiple platforms will be essential, not only for better estimation procedures for differential expression but for other purposes as well.

### Need for standardization in public databases

Public databases are an important resource for investigators to consider. With the stores of chips accessible online, valuable information concerning genes can be compiled and used to supplement new studies and avoid duplication of effort. This methodology would be improved by modification to the databases. Mainly, it is very important to start gathering the raw files instead of processed files. With Affymetrix chips, for example, .cel files should be stored, so that they can be processed using the latest methodology and maintain their usefulness. Already, many of the chips in the GEO database are less useful because they only report values processed through MAS 4.0, an outdated methodology, and comparing these with values generated through MAS 5.0 introduces another source of variation. In addition, the chips should be categorized and sorted according to tissue type, to further facilitate grouping and analysis. These modifications would improve the ability to use previously run chips, thus countering the high costs associated with microarray experiments and enabling the sharing of information to accelerate progress. Thinking about how to take advantage of these databases could provide further improvements to methodologies and enable more tools to be used to study gene functions.

## Conclusions

This work proposes that value lies in pooling information from previous studies. Specifically, gene-specific information can be collected from public databases housing many chips, supplementing new studies and ensuring more reliable results. We show that compiling information from databases provides us with a different and potentially more accurate estimate of gene-specific variance, improving our differential expression analysis in small samples. In addition, because this improvement seems largely independent of the method of analysis, we are able to combine it with regularization in a voting method, leading to superior results. There were particularly strong improvements in the identification of the smallest groups of most differentially expressed genes, which would probably be deemed most important by an investigator as they are easiest to validate. Overall, the scale of the improvement is significant, as it allows investigators to halve their costs in some cases and still retain similar accuracy. The same approach can also be formulated in other settings, especially in the Bayesian framework in which priors for the gene variances may be estimated from previous datasets. Furthermore, as public databases are steadily growing in size, we expect refinement of this method to deliver greater success in the future. Regardless of what method an investigator might use, public databases are clearly a useful source of information and should prove useful in supplementing microarray studies.

## Materials and methods

Because the very nature of public datasets implies that many of the chips have been generated and processed in different manners, standardization of the data is paramount. To maintain comparability, the chips were filtered to remove any chips processed with an algorithm other than MAS 5.0. After removing other unusable chips (such as duplicates and abnormally processed chips) 471 HG-U95A chips remained.

Normalization of all of these chips is crucial, in order to guarantee that scales are similar. In an effort to preserve the general characteristics of each chip, conforming their scales while allowing for some chip-by-chip variability, experiments with multiple methods of normalization were carried out. The two major types included normalizing the trimmed mean and trimmed variance of each chip and using percentage ranks instead of numerical expression levels. In the first case, all of the data points were adjusted to align the mean and variance of the middle 90% of values. In the percentage ranks method, the values were assigned percentiles, removing most normalization effects. In addition, a scale was generated that related the percentile with the average rank change of that percentile. The average rank change for a gene in the middle of the scale was significantly larger than the average rank change at either extreme. This scale was used to adjust the variances on the basis of the rank. Because the results from both normalization methods were fairly similar, only the results of the trimmed mean, trimmed variance experiments are reported here.

After all of the chips were normalized, the gene-specific variance was calculated. These variances were calculated in two separate ways, using a global variance and a pooled variance:





where, for each gene, *x*_*ij *_is the expression level of array *i *in experiment set *j*;  the mean in the experimental set *j*;  is the mean in all arrays; *D *and *D*_*j *_contain the indices for the experimental sets and the arrays in the *j*th set, respectively. The global variance tends to reflect the degree that a gene may vary between different tissue types and diseases while the pooled variance reflects the degree that a gene tends to vary within each experiment. The global variance proved slightly more effective in the cancer dataset, while the pooled variance was more effective in the muscular dystrophy dataset. This seemed to be correlated to the composition of our GEO background datasets. Our set of 471 GEO chips contained 210 cancer chips but only 42 muscle chips. Because a large proportion of the total chips were cancer chips, a global variance may have more accurately represented the information in the whole dataset. However, because so many of the GEO chips were non-muscle, readjusting them into a pooled variance format may have provided a better gene-specific assessment of general expression. We further filtered the variance calculations to eliminate artifacts created by improperly processed chips along with biases from experimentation (that is, if a certain experiment produced uniformly high values for a specific gene). Thus, the highest and lowest 10% of values for each gene across the full set of GEO arrays was trimmed off for the variance calculation. The 10% parameter was chosen experimentally, by tracking how stable variance calculations were as various percentages were trimmed.

The statistical properties of these variance estimators are difficult to show rigorously. If the samples from the GEO datasets can be assumed to come from the same population as those in the current study, the estimators should be unbiased and the proposed test statistic should behave as *N*(0,1) asymptotically. Because the GEO data are an aggregate of many experiments under different conditions often processed differently, we cannot assume the same underlying distribution in general and hence we do not know if the estimators necessarily approach the true variance. However, these estimators appear to be reasonably good approximations to the 'true' variance as demonstrated by the numerical results, and they certainly perform better than estimates based only on the current data.

A master list of the most differentially expressed genes in the dataset was determined by *t*-test analysis. Then, the various methods were compared with each other through a process of subsampling to determine how accurately the results reflect the master list. After two samples from each group were randomly selected, all the genes were filtered out that did not have an expression level above 100 in any of the samples. The goal was to lower false positives among the non-GEO methods, as their results could easily be influenced by small expression levels that by chance ended up with virtually no variance and thus were assigned large *t*-statistics. After processing in this way, the top genes derived using the *t*-test, the regularized *t*-test, and the GEO-adjusted method were compared with the master list to determine their effectiveness. This subsampling procedure was repeated 500 times for each experiment, and the results were averaged. These methods are outlined in the Results section.

Although the GEO-adjusted method was superior to both the *t*-test and the regularized *t*-test, the greatest success was found by averaging the results of the regularized *t*-test method and the GEO method. By averaging the ranks that we receive using GEO and using the regularized *t*-test set, our results are improved concerning our most important genes. This is not seen when the results of the simple *t*-test and GEO are combined, because the lists produced by the simple *t*-test are simply too inaccurate. However, as GEO and regularized *t*-test produce lists that are similar in quality, yet different in nature, a boost can be obtained by averaging the lists. Because using just the GEO variances ignores some of our experimental data and using just our experimental variances ignores global data, it seems that an averaging or voting procedure is a superior way to optimize results. In particular, the system that we used averaged 75% of the value of the lower rank (nearer the top of the list) with 25% of the value of the higher rank. A final score was obtained by combining the results from each method in this way, and the genes were re-ranked on the basis of this score. By using the 75%/25% ratio, genes that have a particularly high ranking on one of the methods are given slightly more importance than genes that have average rankings in both methods. Empirical testing of a number of combinations showed that the 75%/25% combination returned superior results, although all combinations experimented with returned results that were better than either method alone.
